# Factors Associated With Coverage of Health Insurance Among Women in Malawi

**DOI:** 10.3389/frhs.2022.780550

**Published:** 2022-05-13

**Authors:** Margaret Chauluka, Benjamin S. C. Uzochukwu, Jobiba Chinkhumba

**Affiliations:** ^1^Department of Health Systems and Policy, Kamuzu University of Health Sciences, Blantyre, Malawi; ^2^Department of Community Medicine, College of Medicine, University of Nigeria, Nsukka, Nigeria

**Keywords:** health insurance, women, health care equity, gender disparities, Malawi

## Abstract

**Introduction:**

Access to healthcare for the vulnerable groups including women has long been a theme encouraged worldwide from the first general assembly on health to the current sustainable development goals. Despite many countries having a free public healthcare system, there are inequalities in access to care and significant out-of-pocket spending, pushing most women into poverty against the principles of universal health coverage. Coverage of Malawian women with health insurance is poor; thus, there is limited cushioning and high risk of poverty, as women bear costs of care as primary caregivers. There is need to explore determinants of coverage of health insurance among women in Malawi to inform health policy.

**Methodology:**

This study was quantitative in nature, using cross-sectional secondary data from the 2015 to 2016 Malawi Demographic and Health Survey involving women aged between 15 and 49 years. We assessed factors associated with insurance coverage by comparing women with and without insurance schemes using binary logistic regression. Analysis was done using STATA statistical package version 13.

**Results:**

The analysis included a total of 24,562 women with a mean age of 28 SD (9.3). Of these cases, 1.5% had health insurance. High education attainment, occupation, and wealth were significantly associated with health insurance ownership, with all having *p*-values of < 0.01. On the other hand, a woman's residence, marital status, and who heads a household were not associated with ownership of health insurance significantly.

**Conclusion:**

Education, occupation, and wealth have a key role in influencing a woman's choice in owning health insurance. This informs policymakers and health insurance providers on how best to approach women's health financing and factors to target in social security programs and health insurance products that speak to women's needs and capacity.

## Introduction

Universal health coverage (UHC) is fundamental to healthy living of citizens in any country (1). Increased coverage of health services has been shown to improve health indicators and contribute to stronger economic development (1). As per the Alma Ata Declaration, all the citizens should have equal access to healthcare as a right not a privilege (2). Universal health coverage ensures that people access quality healthcare they need without suffering from financial hardships (3). This is significant because a country without healthy citizens loses out on a healthy productive labor force to propel its development activities. Essential health coverage for women is vital because women play a crucial role in development. Bloom et al. attest that healthier women are more likely to participate in the formal labor market and, thus, they face higher opportunity costs of having children (4). As a result, investments in women's health lead to a substitution away from having many children toward having fewer, better-educated children (4), which translate to productive demographic dividends. In addition, investments in women's quality healthcare improve child survival, as women are able to access pre- and postnatal care timely, as well as necessary vaccines, which improve survival rates of children (5). This ensures continuity in future development dividends.

Health insurance is one strategy promoted by the WHO to minimize catastrophic financial hardships when accessing healthcare and ensuring UHC. Global health insurance coverage data often shows that women are disadvantaged. Although women often have greater health needs through their life cycle than men, they are less likely to have insurance, due to economic inequalities and, therefore, have lower ability to pay for services (6). Witter et al. argue that in fact women ought to be the beneficiaries of cross-subsidies from the more privileged groups in society in accessing health services because of all the social cultural, social economic discrimination, and other inequalities that hinder them from accessing quality healthcare (7). Nevertheless, one would argue that with the economic downturn across the globe, the “privileged” is getting fewer and it is unsustainable to work on such a principle for subsidizing healthcare for women considering the methods used such as increased taxation and aid. It is, therefore, important to critically look at health financing alternatives, especially health insurance coverage to ensure that women have access to quality healthcare without risk of incurring heavy financial losses.

## Sub-Saharan Africa Context

In the case of sub-Saharan Africa, most systems be it political, economic, and health were inherited based on colonial operations. For example, health systems inherited by many countries are organized and financed by governments, which provide facilities, personnel, and other inputs (8). By the 1980's, however, many sub-Saharan countries were highly indebted, which led to economic downturn in many of the countries (9). As part of structural adjustment programs, many governments had to cut public spending on infrastructure and services, including healthcare and education (10). Consequently, many African governments ceased subsidies to public services and began implementing various cost-recovery measures in public services (10), which, in turn, led to introduction of out-of-pocket (OOP) expenditures for healthcare (8). Since then, out-of-pocket expenditures have remained high in many sub-Saharan countries (8). With gender disparities in education, economic opportunities, and wages across sub-Saharan Africa (11–13), it has been shown that women's ability to pay for healthcare and make informed decisions affecting their health are heavily compromised (14–16). Amu et al. (17) argue that access to health insurance for women in this region correlates with education. Education enables women to protect their own health and to seek appropriate healthcare when they are ill (17). Thus, being covered by health insurance enables them to avoid catastrophic health expenditures that they may, otherwise, have faced had they made huge out-of-pocket payments when they fell ill in the absence of health insurance (17).

In addition, Gysels et al. (18) and other studies bring another important aspect in healthcare that women shoulder. They present that caring for patients does not only bring financial burden, but it also has a physical, emotional, and social burden for carers (18–20). Having insurance at the point of sickness provides an opportunity for care for the patient, but also cushions the caregivers and family, as they contribute toward transport and providing appropriate food. In addition, there is evidence of psychological impacts of caring for patients such as loneliness, depression, and isolation, which are lessened by reducing the pressure of out-of-pocket payments, especially for women caregivers, who were found to be among the group that often provides care (18–20). With a high burden of chronic diseases such as HIV, malaria, TB, and high rates of maternal mortality coupled with poverty as a hindrance to access healthcare, the need for health insurance as a means of granting access to healthcare for vulnerable women populations cannot be overstated (8).

## Malawi Context

In the Malawian context, the health sector is disaggregated into three tiers, namely, primary, secondary, and tertiary (21). This is a system adopted from colonial times and has been undergoing reforms with time. Health services are provided by public, private for-profit (PFP), and private non-profit organizations, with government providing most (63%) health services (21). The PFP sector is small, but growing, and includes a range of private hospitals and clinics that vary from group to solo practices (21). The major faith-based providers are organized under the Christian Health Association of Malawi (CHAM), which provides ~37% of all the health services and charge service fees (22). Despite its dominance in providing free healthcare, government's increasingly insufficient health expenditure allocation to the health system has led to failure in state service delivery, shifting the burden of access to effective healthcare to the private healthcare providers (8). Although per capita total expenditure on health is better than most low-income countries in the sub-Saharan region at US$39 (23), it is still lower than what is required for Malawi to provide essential healthcare as outlined in the national Essential Healthcare Package (23). Consequently, only 44% of health facilities in the country are able to comprehensively deliver the health services under this package (23). Failure to provide adequate medicines and health workers, insufficient equipment, and poor access to emergency services (22) have necessitated the use of out-of-pocket expenditure to access quality healthcare. As a result, household's contribution to total health expenditure has consistently increased over the past decade, currently standing at 11.2% (24) and likely to continue on this trend, which has implications for equity of access to healthcare if the increased spending is borne by poor households and especially women who constitute the majority of the poor, lag in economic productivity, and face wage inequalities (25). Furthermore, Abiiro et al. (22) found that despite having free services, Malawians are having to spend out-of-pocket on illegal and informal payment to access medical services, which put people in financial risk that most often prevent them from access to care (22).

Currently, Malawi has no social medical insurance in place. However, with increasing pressure to provide adequate healthcare, introduction of a social health insurance has been a theme of reform by the Malawi government as an alternative to health financing (26). Learning from other forerunners in the region, introducing a tiered system that provides health insurance products according to affordability and sectors would help to increase access to quality care by most of the population (17). Having a formal system would also help households plan their income better as opposed to random charges from illegal and illicit dealings. Although still under discussion, it is worth considering as the population is already under strain from OOP expenditure and illicit payments in accessing healthcare in government facilities and it is estimated that over half of the population is being pushed into poverty due to these catastrophic financial outlays (27). As indicated above, having health insurance at the point of access reduces the dilemma of whether to spend on healthcare and get further impoverished or forgo the care, prolonging the illness and risk being unproductive or able to work. In addition, insurance provision introduces competition in healthcare service provision with government operating majority of facilities, which may lead to lowering the costs of providing care for individual patients with resultant positive effect on household income (28).

The private medical insurance industry is relatively small covering 2% of the male population and only 1% of the female population (29). These are mostly from formal labor market as it is easier to collect premiums vs. the informal sector where wages are low, with unorganized structures, which makes it challenging to collect premiums (30). Nevertheless, most of the population belong to this sector particularly women and are more vulnerable to financial shocks; hence, we cannot continue to ignore the need for medical insurance for the vast majority of the country.

Although statistically the difference in coverage for men and women is small, study has shown that it is mostly women who bear the most burden of disease and suffer the effects of ill health and as such are more vulnerable without health insurance (31). In particular, it has been noted that poverty, economic inequalities, education, and culture impede women in ownership of health insurance (17, 31, 32). The recent Integrated Household Survey (IHS) shows that although there are less female-headed households in Malawi (25%), female-headed households have more household members and a higher dependency ratio (1.6) than male-headed households (1.1) (33). With the high levels of poverty, it can be implied that women have a high burden of managing family members' welfare, health inclusive. In many sub-Saharan countries, women (mothers in particular) are critical because they bear most of household responsibilities and it is their health and wellbeing that largely determine the child survival and wellbeing (34) underscoring how beneficial health insurance would be in improving not only access to quality health for mothers, but also their children.

Evidence exists on willingness-to-pay for health insurance even from rural communities and preferences for microhealth insurance, as well as on private demand (16, 22, 35, 36). Abiiro has shown that formulating products that speak to the context of Malawi, such as co-payments, transport facility, and reproductive health just to mention a few, would increase enrollment into a social health insurance scheme (SHI) and suit affordability of households (22, 36). Similarly, the WHO encourages low-income countries such as Malawi to cover “essential health services” and key infectious diseases according to the cultural contexts and economic realities to ensure fairness and equity (37). Although there have been such studies, there is limited focus on women coverage specifically despite that they are in majority and face extreme inequalities in health; hence, this study builds on this pool of knowledge with a focus on insurance coverage for women. The aim of this study is to explore demographic and socioeconomic factors associated with coverage of health insurance among women in Malawi that will provide lessons and knowledge that address women's needs toward improved financing alternatives in the health system.

## Conceptual Framework

**Figure 1:**
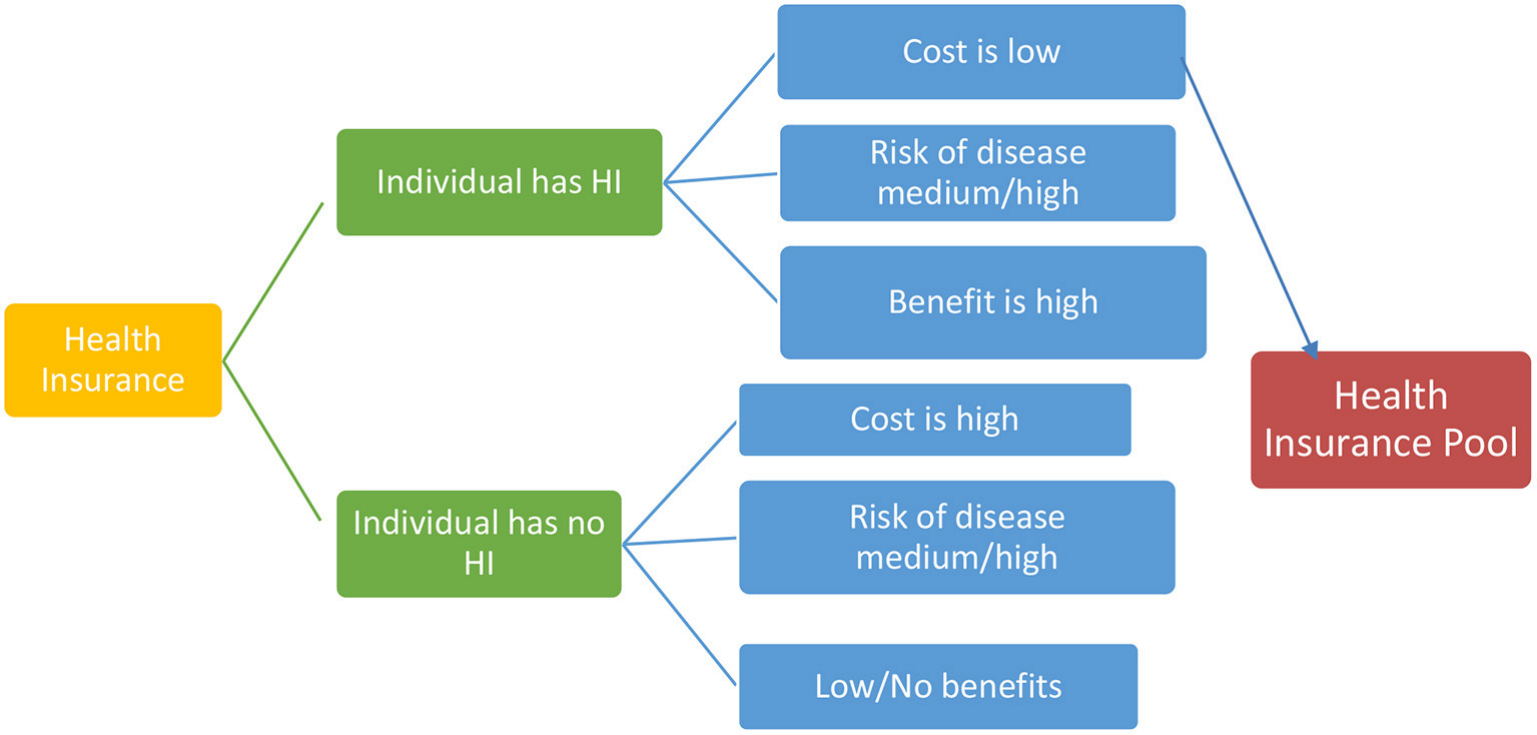
A simple representation of utility choice in pooling resources for health insurance choice (adapted from LSF Microeconomic Policy Analysis, New York: McGraw-Hill; 1986).

This study dealt with two subject matter where individuals exercise choice to have health insurance or not and factors enabling or impeding that choice. The choice to have health insurance or not is embedded in welfare economics. Welfare economics theory tries to answer if we can derive a social preference from the preferences of individuals (38). This is a theory of uncertainty, which predicts that individuals are generally risk averse and where faced with risk, individuals would like to insure against all the perceived forms of risks (39). The theory also explains that everyone strives to maximize the expected value of a utility function that is how much benefits will be derived from spending on health insurance in the case of this study. Risk aversion entails that individuals have a diminishing marginal utility of income. Since health risks for different individuals are basically independent, pooling them reduces the risk to the insurer to relatively small proportions (39) Figure 1 shows a graphic model of the theory.

**Figure 2:**
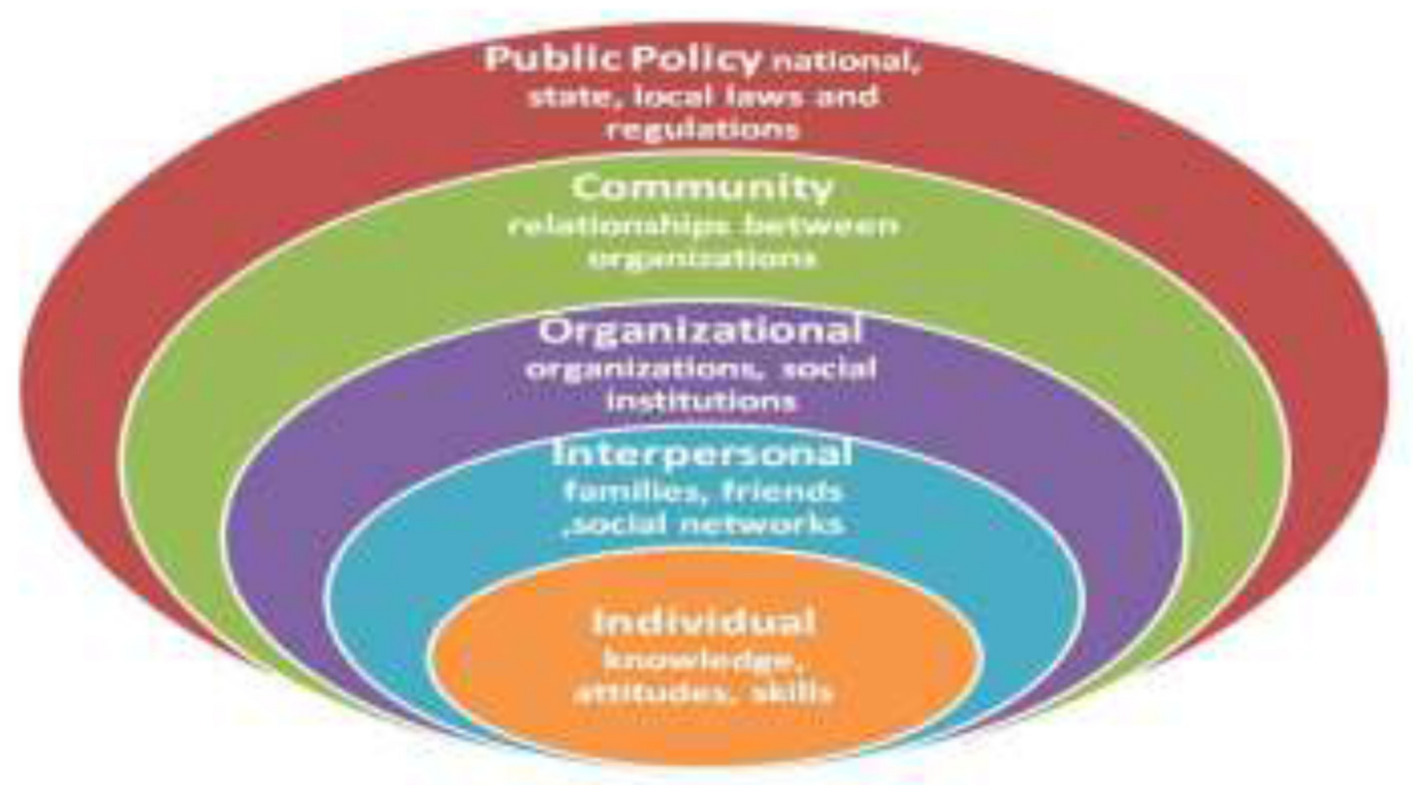
A representation of the social ecological model (adapted from centers for disease control. Available from the Centers for Disease Control and Prevention (CDC) (42).

On the other hand, welfare economics cannot fully explain enabling and impeding factors for this choice, which bring us to the social ecological model (40, 41). This model helps to understand factors affecting behavior and also provides guidance for developing successful programs through social environments (40, 41). Social ecological models emphasize multiple levels of influence (such as individual, interpersonal, organizational, community, and public policy) and the idea that behaviors both shape and are shaped by the social environment. For this study, we look at individual behavior, environmental, economic, and social factors as independent variables and their interaction with the choice an individual make, i.e., to purchase a health insurance or not. Figure 2 shows a representation of the social ecological model.

## Methodology

This study used cross-sectional secondary data from the 2015 to 2016 Malawi Demographic and Health Survey collected from all the 28 districts of Malawi (29). It is an internationally recognized survey conducted every 5 years on health indicators in Malawi with an excellent track record in robust methodologies used. Full details of methods can be accessed at DHS Methodological Report series. Looking at the subject matter, ownership of health insurance, the literature informed selection of independent variables. As mentioned above, a woman's education, the area she resides in, her age, marital status, the work she does, and her level of wealth are shown to be associated with ownership of health insurance (17, 31, 32, 43, 44). In addition, selection of the variables also looked at the context relevant to the sub-Saharan region and Malawi in particular, since we used secondary data; the final variables entered into analysis were also based on data available in the DHS data set. The variables and their definitions are shown in Table 1.

**Table 1:** Dependent and independent variables, their definitions, and measurements.

**Independent variables**	**Definition and measurements**
Age	Age is a continuous variable from 15 to 49 years
Residence	Place of residence categorized as 1 if respondent resides in urban,0 if in rural
Education	Categorical variable, defined as 0 if no education, primary, 1 if secondary, higher
Occupation	A womans occupation defined as 0 if informal and 1 if formal
Marital status	A womans marital state defined as 0 if not in union (widowed, divorced, separated), 1 if married (living with a partner)
Household head	Household head position defined as 1 if female headed and 0 if male headed
Wealth	Wealth status defined as 1 if poorer;2 if middle class, 3 if richest

### Study Population

All the women aged 15–49 years who were either permanent residents of the selected households or visitors who stayed in the household the night before the survey were eligible to be interviewed.

### Sample Size

The DHS surveys collect data on marriage, fertility, mortality, family planning, reproductive health, child health, nutrition, and HIV/AIDS just to mention a few. Due to the subject matter of the survey, women of reproductive age (15–49 years) were the focus of the survey. Apart from these subjects, the Malawi DHS has other additional modules of interest such as health insurance and in this study, we focus on the sample of women who reported to have health insurance, be it commercial-, institutional-, or employment-linked health insurance. In selecting study population, sample was stratified and selected in two stages. Each district was stratified into the urban and rural areas; this yielded 56 sampling strata. Stratifying is important to reduce bias in outcomes (29). Thirty households per urban cluster and 33 households per rural cluster were selected with an equal probability systematic selection from the newly created household listing. A total of 26,361 women were identified as eligible and out of these, 24,562 women were interviewed and responded to the questions in this module making the total sample size of this study.

**Figure 3:**
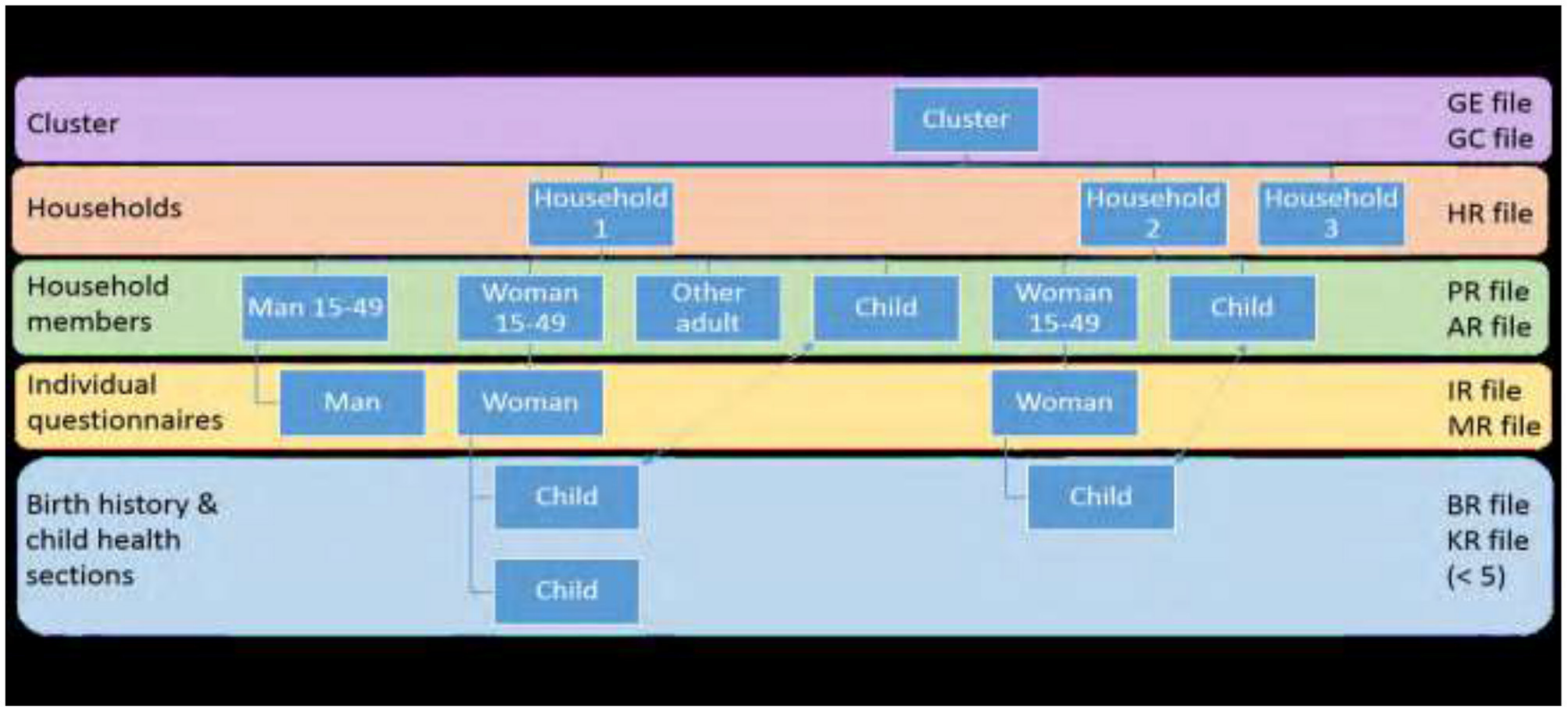
A summary of sampling method for the demographic health survey (Adapted from the Guide _to DHS_statistics DHS-7).

Data were collected by trained enumerators using structured questionnaires. Figure 3 illustrates the sampling process used in the Malawi Demographic and Health Survey.

### Data Collection

Data were collected on women's background characteristics (age, education, religion, etc.), reproduction, contraception, pregnancy, and postnatal care immunization. In addition, the questionnaire gathered information on women's health practices and health expenditure. Data were collected electronically to improve on human error and stored in password protected laptops. For this study, we used the household questionnaire and the women's questionnaire as our data tools with the women's questionnaire as the central focus in this study from the Malawi demographic health survey tools.

### Data Analysis

This study evaluated women in terms of ownership of health, showing percentages of the two categories (ownership or not) entered as a dichotomized variable in the model. The outcomes of interest were weighted and frequencies of each variable conducted to show the characteristics within the variables using two-way tables with measures of association such as the chi-squared test. A detailed process of weighting of variables can be found in the Methodological Report series (DHS) Methodological Report series.

A woman's age was included as a continuous variable in the final module and categorical in the bivariate analysis because the association between age and health insurance may be non-linear. Household head and residence were a dichotomized variables (1, 0), whether head was male or female and rural and urban, respectively. Education for a woman is a self-reported measure reflecting the highest education level at the time of the survey and grade within that level. In this analysis, we used a recoded variable of a woman's education categorized into the two groups: primary education and higher education. Marital status at the time of the survey was coded as never married, currently married, living with partner, widowed, divorced, and separated; however, for this analysis, the categories were collapsed into not in union and married. Occupation included all the occupations, paid and unpaid at the time of survey defined as formal and informal for this analysis. Wealth quintile index was constructed based on data collected in the Household Questionnaire. This questionnaire included questions concerning the household's ownership of a number of consumer items such as a television and car, dwelling characteristics such as flooring material, type of drinking water source, toilet facilities, and other characteristics that related to wealth status. From this information, women were assigned to the various categories of poorest, poorer, middle class, richer, and richest (45). In this analysis, we used the categories of 1 if poorest, poorer, 2 if middle class, and 3 if richest class.

To analyze the overall model, we used the logistic regression method in STATA version 13. This model allows us to evaluate multiple explanatory variables by extension of the basic principles. All the variables were entered as continuous variables using a backward elimination process, removing variables that did not contribute to the regression equation. We used the Pearson's chi-squared test to show goodness of fit of the model. *p*-values were used to present significance of covariates. In addition, the odds ratio (OR) shows the likelihood or probability of occurrence given a value. In this case, likelihood of having health insurance given the various independent variables. The model is expressed as:


(1) $$\begin{array}{r} P=\frac{e^{a+bX}}{1+e^{a+bX}} \end{array}$$

Table 1 shows the summary of independent variables, their definitions, and measurements.

## Results

**Figure 4:**
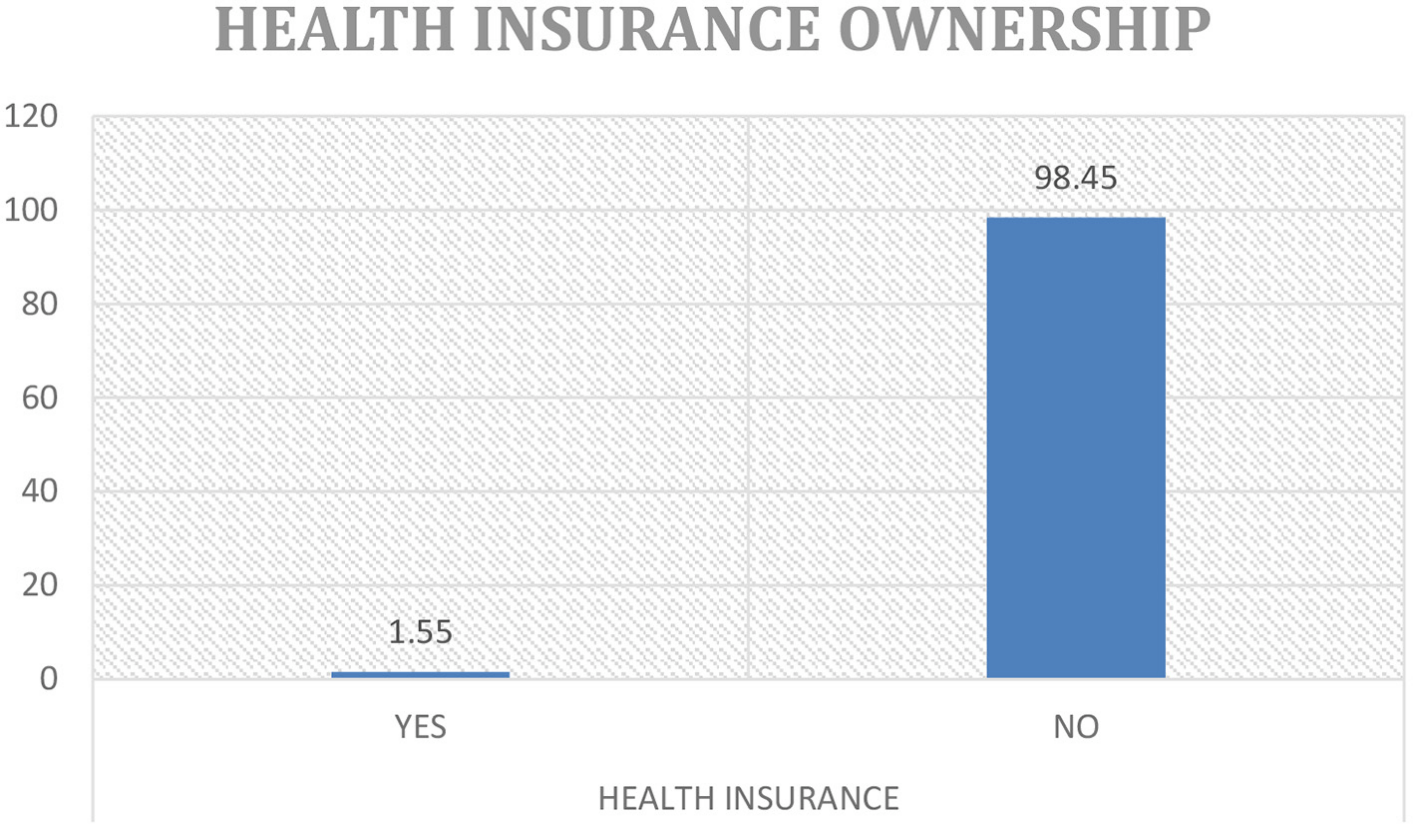
A representation of health insurance ownership.

Out of 24,562 cases in the dataset, 1.5% (380) of women had health insurance as Figure 4 illustrates. Of these 380 women, 87% had employee linked coverage and the remainder accessed commercial health insurance products. As Wang et al. (46) echoes this result is congruent with many low middle income countries. It is a common trend to see low levels of health insurance ownership; it was, therefore, imperative to focus on this aspect due to the lack of empirical data demonstrating the state of health insurance coverage and women in particular and its potential effects on the use of healthcare services in the country. It was also important to shed light on the 97% of women excluded. It provides a basis for further study qualitatively, targeting more than women available at home at the time of survey as was the case in the DHS, including those in formal and informal workplaces to get a true reflection of the problem. The women in this analysis had a mean age of 28 (SD 9.25) years, 65.7% were married, and most (81.7%) were from rural areas. Predominantly, households were male headed (70.5%) compared to 29.5% headed by women. Most women fell in the poorer (38.4%), middle class (31.92%) compared to 23.6% in the richest category, respectively. More sociodemographic features of the participants are given in Tables 2, 3.

**Table 2:** Demographics of the sampled population.

**Variable**	**Frequencies**
**Age**	24,562 (SD 9.3)
15–19	5,273 (21.47%)
20–24	5,094 (20.7%)
25–29	3,976 (16.2%)
30–34	3,648 (14.9%)
35–39	2,988 (12.17%)
40–44	2,022 (8.23%)
45–49	1,561 (6.36%)
**Occupation**	
Informal	21,600 (87.9 %)
Formal	2,962 (12.1 %)
**Household head**	
Male	17,316 (70.5%)
Female	5,810 (29.5%)
**Residence**	
Urban	4,495 (18.3%)
Rural	20,066 (81.7%)
**Education**	
Primary	23,820 (96.98%)
Higher	741 (3.02%)
**Wealth**	
Poorer	9,437 (38.42%)
Middle	9,314 (31.92%)
Richest	5,810 (23.66%)
**Marital status**	
Not in union	8,431 (34.3%)
Married	16,130 (65.67%)

**Table 3:** Relationship between health insurance and covariates.

	**Covered by health insurance (*N* = 24,561)**			
	**No (*****N*** **=** **24,181)**	**Yes (*****N*** **=** **380)**	**Chi-square**	***P*** **-value**
**Residence**				
Urban	5,039 (96%)	208 (4%)	0.000	0.18
Rural	19,186 (99.3%)	129 (0.7%)		
**Education**			0.000	0.00
Primary	23,658 (96.98%)	54 (3.02%)		
Higher	567 (81.7%)	127 (18.3%)		
**Household head**				
Male	17,026 (98.6%)	247 (1.4%)	0.230	0.23
Female	7,199 (98.8%)	90 (1.2%)		
**Wealth**				
Poorer	8,697 (99.87%)	11 (0.13%)	0.000	0.00
Middle	9,364 (99.56%)	41 (0.44%)		
Richest	6,164 (95.6%)	285 (4.4%)		
**Marital status**				
Not in union	8,502 (98.5%)	108 (15%)	0.082	0.24
Married	15,723 (98.6%)	229 (1.4%)		
Occupation	24,225 (77%)	337 (23%)		0.00
Informal	21,444 (99.3 %)	156 (0.72 %)		
Formal	2,781 (93.89 %)	181 (6.1 %)		
**Age**				0.00
15–19	5,235 (99.28%)	38 (0.72%)		
20–24	5,038 (98.9%)	65 (1.10%)		
25–29	3,909 (98.3%)	67 (1.69%)		
30–34	3,573 (97.9%)	75 (2.1%)		
35–39	2,945 (98.6%)	43 (1.44%)		
40–44	1,987 (98.3%)	35 (1.73%)		
45–49	1,538 (98.5%)	23 (1.47%)		

Table 2 gives the demographics of the sample and their frequencies.

Table 3 describes weighted frequencies and percentages of independent variables in relation to the dependent variable. It shows that younger women aged 15–19 and 20–24 years had less likelihood of being covered by health insurance with only 7 and 1.1% coverage, respectively. However, those aged between 25–29 and 40–44 years had similar coverage with 1.7% of women in both the age groups covered by health insurance. Those between the ages of 30–35 years had the highest coverage of 2.1% and finally those in the final bracket of 45–49 years had a coverage of 1.5%. Predominantly, living in the urban area, a woman had a higher chance of being covered by health insurance at 4% coverage than women in the rural area. In addition, women with no education and a primary education had the lowest coverage 3% and compared to those who had attained higher education who had the highest coverage of 18.3% both showing a positive association with owning health insurance.

Coverage of health insurance in male-headed households was 1.4% points higher than for female-headed households. The result also showed that the more money a woman had, the more likely she would own health insurance with the poorest and poorer wealth categories having the least coverage at 0.1% compared to the richest class (4.4%). Although over 98% of women reported to have been in some form of occupation, only 6.8% were covered by health insurance. Finally, those that were not in any union had the higher coverage than married, widowed, and divorced combined. The results showed that household head is not associated with a woman owning health insurance.

Table 4 shows the results using logistic regression model. The odds ratio was used to show likelihood of having health insurance given the independent variables. For every year of age, the odds of owning health insurance increases by a factor of 1.02, as shown in Table 4. Nevertheless, moving to an urban area of residence increased the odds of having health insurance compared to rural areas (95% CI: 0.5770–0.9645). Similarly, wealthy women had a positive likelihood to own health insurance; therefore, improving wealth level would increase the chances of a woman having insurance for healthcare. High education attainment also increased positively association with health insurance ownership. A male household head is likely to own health insurance than a female and those married also stand better odds of having health insurance than those not married (1.4 more times). Having an occupation gave a woman twice the odds of having health insurance. Education, wealth, and occupation were significant at 95% CI, while age, residence, marital status, and household head were not significant.

**Table 4:** Results of the logistic regression analysis model.

***N*** **=** **24,562** **Prob** **>** **chi**^**2**^ **=** **0.0000** **Pseudo** ***R***^**2**^ **=** **0.2345** **Pearson Chi**^**2**^ **(1,541)** **=** **1,461.89**					
**Health insurance ownership**	**Odds ratio**	**Std. Err**.	***z***	***P*** **>|** ***z***	**[95% Conf. Interval]**
Age	1.00839	0.0073803	1.14	0.254	0.9940287 – 1.02296
Residence	0.7936482	0.104363	−1.76	0.079	0.6133339 – 1.026973
Education	5.606138	0.7868402	12.28	0.000^*^	4.257894 – 7.381299
HH Head	0.9270029	0.1365027	−0.51	0.607	0.6946088 – 1.237149
**Wealth**					
Middle	3.03394	1.033773	3.26	0.001^*^	1.555865 – 5.916192
Richest	15.97973	5.190536	8.53	0.000^*^	8.454384 – 30.20346
Marital status	15.97973	0.1900378	1.70	0.089	0.9624323 – 1.717829
Occupation	2.398547	0.3202295	6.55	0.000^*^	1.846309 – 3.115961
_cons |	0.0010086	0.0003848	−18.09	0.000	0.0004776 – 0.0021304

* *represents significance of the variables*.

## Discussion

This article explored demographic and socioeconomic factors that are associated with ownership of health insurance among women in Malawi. It shows that demographically there is a youthful population of women most of whom are in the rural areas. Predominantly, most women have either a primary or secondary education, but are not educated for high technical skills and, hence, are unemployed or working in the informal sector contributing to the higher percentages in poorest, poorer, and middle class categories. As aforementioned, a woman's decision to own health insurance is not only influenced by the health risks. As the social ecological model describes, we see that there are individual and systemic factors influencing this choice. For example, most women have primary or no education, which is a result of individual choice, but also systemic in cultural nuances and access to education facilities. We note that occupation and wealth are not just a function of an individual's choice, but also on the economic environment of a society and equality in provision of opportunities just to mention a few. We explain these factors in detail.

### Demographic Variables

Age was not a significant variable in the analysis. It is interesting to note, however, that women in the ages of 30 to 45 years have higher health insurance coverage as compared to those below 30 years despite the under 30's being a majority. This speaks to demographic disparities in the country, especially in the context where youths, including young women, are unemployed and vulnerable to poverty. However, this result varies with findings of age significance from Kirigia et al., Wang et al., and Aregbeshola et al. arguing that it is a common state in many African countries such as Nigeria, Namibia, Rwanda, and South Africa just to mention a few (32, 43, 46).

It was also important to note that women living in the urban areas were more likely to own health insurance than those in the rural areas. The literature on this is mixed: while a study in South Africa showed that there were no significant differences (32), others have argued that it is a significant determinant (47–49), noting that in many African countries majority of the population reside in the rural area with low incomes and lower levels of education, which could explain the likelihood of those in the urban areas to own HI than those in the rural areas.

In terms of who heads a household, we found that the relationship is not significant in owning health insurance similar to findings by Kimani et al. (48), but unlike the findings by Aregbeshola et al. (43). Possible reason for this could be the fact that both the men and women appreciate the need for health insurance for their households irrespective of who heads the household.

Marital status was not a significant determinant of a woman's choice to own health insurance in the multivariate analysis. Those who were not in any union had more likelihood to have health insurance than those married. Nevertheless, other studies (32, 47) show an association between married women and owning health insurance arguing that married women are beneficiaries from their partners and also the combined income increases disposable income in the household to afford health insurance. In our context, more women were unemployed and predominantly in low paying work and this might explain our results.

### Socioeconomic Variables

Education was significantly associated with health insurance ownership. Those with higher education were the most reported to have health insurance (18%) compared to those with primary or no education who were a tiny fraction in the categories with only 3% reported to have health insurance. As Wang et al. and others (32, 48, 50) have found, education was a key determinant in health insurance in many developing countries, especially for women. In view of this, investments in education not only have to improve, but also target women, especially in the rural areas providing the necessary infrastructure and resources while ensuring systemic and cultural gender barriers are minimized for young women to attain education. Wealth was also significant in determining a woman's choice in owning healthcare. Those in the richest category were more likely to own health insurance than those in the poorest, poorer, and middle-income categories a result which is consistent with other literature (17, 50) suggesting that the design of health insurance systems works in favor of the rich and not necessarily the poor. The implication is that in our context, government needs to be more meticulous in the design of a social health insurance system that will respond to the needs of the poor, for example, disaggregating premiums with the rich paying more for select services to subsidize lower premiums for those in the poor categories.

In addition, a woman's occupation showed significance in determining health insurance ownership in the analysis. Women in highly skilled jobs such as administrative and commercial managers science and engineering professionals, teaching, health professionals, and information technology technicians had a more likelihood of having health insurance than those in low-skilled jobs such as farmers, sales, and personal care workers a finding resonating with a study in Namibia (51).

It is interesting to note that although women in high-skilled jobs had higher likelihood to health insurance ownership, there are variations especially in sectors dominated by men such as legal, corporate heads, and armed forces. Women in such areas showed lower insurance coverage probably because they are few. In addition, for those in low-skilled jobs, it is surprising that women working in unskilled but risky jobs such as production, protective services, construction, and machinery operations just to mention a few have low health insurance coverage. Government can ensure policies set on gender equality are well-mainstreamed and enforced at all the levels to create environments where women get equal opportunities and equal pay in the formal sector. The current Malawi Growth and Development Strategy (MGDS) and national gender policies all have set strategies to achieve gender equality and improve womens' access to healthcare though both require political will and financial investments to materialize. Furthermore, policymakers can take advantage of cooperatives, associations of various sections in the informal sector such as the National Association of Business Women (NABW), Malawi Union for the Informal Sector (MUFIS), and the Small and Medium Enterprises Development Institute (SMEDI) just to cite a few, to disseminate information on health insurance, introduce mode of payments, and ensure accountability to ensure that more women are reached in the informal sector.

## Conclusion and Contribution to Field

Universal health coverage is a right not a privilege and in order to achieve inclusive universal health coverage, aspects of how healthcare is financed must be critically addressed, especially for vulnerable populations such as women. As presented above, health insurance provides a cushion for women to access healthcare without suffering from financial loss or being driven into poverty (8). It is important to analyze what determines this choice in order to provide women with the right products, but also policies that will support the underlying factors driving this choice such as education, wealth, and place of residence as the results have shown.

As considerations are made for a national health insurance policy, it would be important to address levels of education of women, especially in attaining tertiary education, as this has been shown to be a key determinant for the choice of health insurance. Deliberate efforts need to be made in this area because as much as education is important in economic development, it can only be meaningful where women are healthy and empowered to access quality healthcare.

In addition, looking at the results, there are policy implications in terms of creation of economic growth opportunities and how they are distributed. Currently, most factories and private businesses are concentrated in the urban areas, which limit opportunities for women in the rural areas. Expanding such productive opportunities to the rural areas extends the opportunities to women increasing their ability to earn. This is important in 2-fold, as government, they increase their tax base that can, in turn, provide resources to fund a social health insurance system, considering that a small tax base is one of the challenges in establishing social health insurance (52). On the other hand, with improved incomes, women would be able to afford health insurance premiums and education for their children mainly young women who are disadvantaged from low household income (53, 54) addressing the key factors prohibiting women's ability to have health insurance.

All in all, as part of health systems strengthening, policymakers and stakeholders in health might want to consider building collaborations with other ministries such as economic development, gender, and education to have holistic strategies that address systemic gender inequalities in healthcare and other social determinants of health. As the famous saying goes, a healthy nation is a wealthy nation and we should all strive to attain that state.

### Limitations

The major limitations of this study are that it is based on cross-sectional data from the Demographic and Health Surveys and as such can only infer association. Second, despite having a robust and large sample in the analysis, the findings are not powered to make inferences at district level; hence, generalization at local levels must be done with caution. Nevertheless, the strengths of findings are premised on the robust study design, sampling, and data collected using standard methodologies.

## Data Availability Statement

Publicly available datasets were analyzed in this study. This data can be found at: https://dhsprogram.com/Data/terms-of-use.cfm; https://dhsprogram.com/publications/publication-search.cfm?type=6.

## Ethics Statement

Permission to use the 2015–2016 MDHS data was obtained from the DHS program. There were no other ethical considerations on the part of the researchers, as the dataset is completely anonymized and all the other pertinent ethical issues were handled by the DHS program before and during the survey. This study was also approved by the College of Medicine Research and Ethics Committee (COMREC).

## Author Contributions

MC contributed to concept direction, mentorship, refining, and editing manuscript from BU and JC. All authors contributed to the article and approved the submitted version.

## Funding

Funding for this research comes from World Health Organization in partnership with African Health Economics and Policy Association.

## Conflict of Interest

The authors declare that the research was conducted in the absence of any commercial or financial relationships that could be construed as a potential conflict of interest.

## Publisher's Note

All claims expressed in this article are solely those of the authors and do not necessarily represent those of their affiliated organizations, or those of the publisher, the editors and the reviewers. Any product that may be evaluated in this article, or claim that may be made by its manufacturer, is not guaranteed or endorsed by the publisher.
